# Comparing the performance of the EQ-5D-3L and the EQ-5D-5L in young Portuguese adults

**DOI:** 10.1186/s12955-016-0491-x

**Published:** 2016-06-08

**Authors:** Lara N. Ferreira, Pedro L. Ferreira, Filipa P. Ribeiro, Luis N. Pereira

**Affiliations:** School of Management, Hospitality and Tourism, University of the Algarve, Campus da Penha, Faro, 8005-139 Faro Portugal; Faculty of Economics, University of Coimbra, Coimbra, Portugal; Centre for Health Studies & Research, University of Coimbra, Coimbra, Portugal; Centre for Studies in Language Sciences, University of the Algarve, Faro, Portugal; Research Centre for Spatial and Organizational Dynamics, University of the Algarve, Faro, Portugal

**Keywords:** Ceiling effect, EQ-5D-3L, EQ-5D-5L, Explorative qualitative interviews, Health-related quality of life, Young individuals, I10

## Abstract

**Background:**

Some studies have reported a ceiling effect in EQ-5D-3L, especially in healthy and/or young individuals. Recently, two further levels have been included in its measurement model (EQ-5D-5L). The purposes of this study were (1) to assess the properties of the EQ-5D-5L in comparison with the standard EQ-5D-3L in a sample of young adults, (2) to foreground the importance of collecting qualitative data to confirm, validate or refine the EQ-5D questionnaire items and (3) to raise questions pertaining to the wording in these questionnaire items.

**Methods:**

The data used came from a sample of respondents aged 30 or under (*n* = 624). They completed both versions of the EQ-5D, which were compared in terms of feasibility, level of inconsistency and ceiling effect. Agreement between the instruments was assessed using correlation coefficients and Bland-Altman plots. Known-groups validity of the EQ-5D-5L was also assessed using non-parametric tests. The discriminative properties were compared using receiver operating characteristic curves. Finally, four interviews were conducted for retrospective reports to elicit respondents’ understanding and perceptions of the format, instructions, items, and responses.

**Results:**

Quantitative results show a ceiling effect reduction of 25.3 % and a high level agreement between both indices. Known-groups validity was confirmed for the EQ-5D-5L. Explorative interviews indicated ambiguity and low degree of certainty in regards to conceptualizing differences between levels moderate-slight across three dimensions.

**Conclusions:**

The EQ-5D-5L performed better than the EQ-5D-3L. However, the explorative interviews demonstrated several limitations in the EQ-5D questionnaire wording and high context-dependent answers point to lack of illnesses’ experience amongst young adults.

## Background

The traditional EQ-5D is a generic preference-based measure of health that has five dimensions each with three levels of impairment (EQ-5D-3L) that, together, describe 243 health states [1]. Many agencies that regulate the economic evaluation of drugs and other health technologies advise the use of preference-based instruments as outcome measure in cost-utility analyses [2]. However, previous research showed that the EQ-5D-3L may not be able to fully discriminate different levels of health status among individuals, especially in the healthier part of the measurement rule [3]. Several studies reported the existence of a celling effect in both the general population and different patient groups (e.g. [4–11]). To improve the descriptive richness and discriminatory power of the EQ-5D, the EuroQol Group has recently developed the EQ-5D-5L questionnaire. The EQ-5D-5L retains the original five dimensions of the EQ-5D-3L, but the number of levels in each dimension is increased from three to five [12, 13]. This EQ-5D-5L defines a total of 3,125 states.

Studies that directly elicit preferences from general population samples to derive value sets for the EQ-5D-5L are under development in a number of countries. In the interim, the EuroQoL Group coordinated a study that administered both the 3-level and 5-level versions of the EQ-5D, in order to develop a “crosswalk” between the EQ-5D-3L value sets and the new EQ-5D-5L descriptive system, resulting in crosswalk value sets for the EQ-5D-5L [14]. Crosswalk value sets for the EQ-5D-5L are currently available for the following countries: Denmark, France, Germany, Japan, the Netherlands, Spain, Thailand, UK, USA and Zimbabwe.

Since the recent introduction of this preference-based measure, some articles have been published using the EQ-5D-5L, and it is anticipated that the application of this measure will continue to grow. Some authors have studied the measurement properties of the EQ-5D-5L in patients with cancer [15], with chronic hepatic diseases [16], with other chronic conditions [17] and with HIV/AIDS [18]. However, to our knowledge, there has been no assessment of measurement properties or validation of the EQ-5D-5L in young adults. Given that they usually are healthy individuals, it is expected that they report a significant celling effect in the EQ-5D-3L.

When analyzing new instruments, it is important to study methodological issues that arise when questionnaires are used, namely how questions are linguistically framed. This kind of research has not been done previously with the EQ-5D. Therefore, the goals of this paper were (1) to compare the psychometric properties of the EQ-5D-5L with the EQ-5D-3L in a sample of young adults, aged 30 years or under, (2) to foreground the importance of collecting qualitative data to confirm, validate or refine the EQ-5D questionnaire items and (3) to raise questions pertaining to the wording in these questionnaire items.

## Methods

### Data collection

Students from two Portuguese universities were recruited, according to their willingness to participate in the study. The study was approved by the ethics committee of the Regional Health Authority, Portugal. Participants were informed verbally and in the questionnaire that the study would be published, and written informed consent was obtained by study participants and made available to the Editor upon request. The target population (students) consisted of young and healthy subjects, a cohort in which we expect a higher ceiling effect on the EQ-5D. Respondents filled one single questionnaire form with both the 3L and the 5L Portuguese versions of the EQ-5D, with socio-demographic questions separating both versions. The order of the self-completed paper-and-pencil questionnaires was fixed and was the same throughout the study: first EQ-5D-5L and second, the EQ-5D-3L. This order was chosen according to previous findings that showed that completing the 5L version before the 3L could help avoiding the tendency of respondents of not using the “in-between” level 2 and 4 of the 5L [19]. Data collection took place in April-June 2013 and October-November 2013. The total sample comprised undergraduate and graduate students and therefore individuals aged 17–49 (*n* = 927). The sample used in the study included respondents aged 30 or under (*n* = 624). The remaining sample was used for comparison purposes (*n* = 303). Students were then asked to volunteer to be interviewed about the questionnaires. From those who volunteered, four students were selected to be interviewed individually. The sessions were conducted with two interviewers, in April-May 2014. Sessions were conducted in Portuguese and had an average length of 17 min each. They were audio-recorded and transcribed for analysis.

### Statistical analysis

Sample characteristics were described by computing descriptive statistics for socio-demographic variables. Feasibility was accessed by computing the number of missing values for all five of the 3L and 5L questions and by dimension. Inconsistency of responses and ceiling effect were also evaluated using the methodology followed in previous studies [15–17, 19]. Briefly, inconsistencies were defined when a 3L response and a 5L response were at least two levels away, according to the redistribution diagram proposed by Janssen et al. [17, 19]. The ceiling effect was calculated as the proportion of respondents reporting full health (11111) and the proportion of respondents reporting no problem (level 1) in each of the dimensions [16]. Additionally we also present the absolute and relative ceiling effect reduction. The absolute reduction is the difference between the proportion of “no problem” responses in both measures and the relative reduction is given by [16]: $\frac{ceiling\ 3L- ceiling\ 5L}{ceiling\ 3L}\times 100$.

To complement this analysis, we have also looked into what respondents reported about their health in each instrument. This task started with a general descriptive analysis of the distribution of responses across the dimensions in both instruments. The level of agreement between the dimensions of the EQ-5D-3L and 5L was measured using Spearman’s correlation coefficient. The level of agreement between the indexes was accessed using Pearson’s correlation coefficient and Intraclass Correlation Coefficient (ICC). Additionally we also present Bland-Altman plots for the EQ-5D-5L and 3L by plotting the average value of both indexes (*x*-axis) against the difference between the EQ-5D-5L and 3L score (*y*-axis) [7]. A score below (above) zero would denote that a particular individual had a utility score that was higher (lower) according to the EQ-5D-3L. In addition the upper and lower limits of agreement are also presented (mean difference ± standard deviation of the difference). These limits show how far apart the two measures are more likely to be for most individuals.

Based on the literature (e.g. [15, 17, 20]) known-groups validity of the EQ-5D-5L was tested with the following hypotheses: females and those with a medical condition were expected to have a lower EQ-5D index score [21, 22]. Given the skewness of the distributions, non-parametric tests (Mann–Whitney test for two groups and Kruskal-Wallis test for more than two groups) were used.

The discriminative properties of the indexes were also compared using Receiver Operating Characteristic (ROC) curves, as has been done in other comparisons between preference-based instruments [8, 9]. The performance of the indexes was evaluated against one indicator of health status: reported chronic medical conditions. The reported chronic medical conditions indicator was dichotomized using two cut-off points, regarding the number of health conditions: none versus one or more medical conditions and none or one condition versus two or more conditions. The measure of utility that generated the largest area under the ROC curve was considered the most sensitive in detecting differences in the external indicator. Hypothesis tests were carried out for the purpose of comparing the areas under the ROC curves.

Though the Portuguese value set for the EQ-5D-3L has been recently derived [23], there is no crosswalk value set for the EQ-5D-5L for Portugal or a Portuguese value set for the EQ-5D-5L. Thus in this paper we used the UK value sets for both the EQ-5D-3L and the EQ-5D-5L. All the analyses were performed using IBM SPSS 21.0.

### Cognitive discourse analysis (CODA)

Survey data might be compromised when respondents do not interpret questions in the way researchers expect. Cognitive interviews are used to detect problems respondents have in understanding survey instructions and items, and in formulating answers. The explorative component of this study is a complementary analysis aiming at better identifying components that might be interpreted differently than intended. It applies a strategy called cognitive interviewing by verbal probing relying on Cognitive Discourse Analysis (CODA) of retrospective reports [24]; i.e. we called back four students who had filled in the questionnaire and they were asked to spell out aloud how they had interpreted the various questions. The main emphasis lies on the systematic analysis of both content and linguistic choices and patterns, aiming to identify indicators for specific cognitive phenomena that are of interest for addressing the way *how* some content is expressed or structured in addition to *what* is said in relation to EQ-5D-5L interpretation. As cognitive phenomena is accessed through language, the analysis focuses on linguistic properties of discourse, namely lexical items, such as adjectives, adverbs, pronouns and also voice and modality in order to identify differences between levels of severity and certainty which cannot be fully ascertained by quantitative data. As this component is a complementary analysis of this study we decided to interview solely students whose responses were inconsistent and/or with a ceiling effect reduction, selected from the poll of students who had volunteered to be interviewed. Therefore the number of interviews was small, but acceptable in cognitive terms.

## Results

### Subjects

Table 1 shows a summary of the main characteristics of the study sample, comparison sample and the overall sample, along with values for the Portuguese population aged over 18–30 for which data are available [25]. The mean age of subjects in the overall sample was 25.5 years, whereas the study sample was on average 21.7 years old and the comparison sample about 38.1. As was expected the study sample was predominantly made up of individuals who were single (93.8 %). Table 1 also shows that the majority of the study sample does not have any disease (82.3 %), a value that is very close to the comparison sample and not that different from the Portuguese population (65.6 %) [26]. However it is worth noting that this is a relatively young sample; therefore one might expect a lower percentage of individuals with a medical condition. Regarding the number of medical conditions, there were 83 (13.3 %) respondents with one medical condition and 28 (4.5 %) with 2 or more medical conditions.

**Table 1:** Study sample characteristics and Portuguese general population aged 18 or more

	Sample of individuals aged ≤30 (*n* = 624)	Sample of individuals aged >30 (*n* = 303)	Overall sample (*n* = 927)	PT general population aged 18–30 (*N* = 1.524.869)^a^
Mean age (SD)	21.7 (3.2)	38.1 (6.3)	25.5 (8.1)	24.1 (3.7)
% women	60.4	61.1	60.6	49.6
% single	93.8	49.5	79.4	63.0
% without a chronic disease^b^	82.2	78.9	81.1	69.2
Mean EQ-5D-5L (SD)	0.896 (0.119)	0.889 (0.133)	0.894 (0.124)	n.a.
Mean EQ-5D-3L (SD)^c^	0.919 (0.114)	0.907 (0.123)	0.915 (0.117)	0.758 (SE-0.006)
Mean EQ-5D VAS (SD)^c^	84.7 (12.1)	82.3 (13.6)	83.9 (12.7)	74.9 (SE-0.504)

*SD* Standard deviation, *SE* Standard Error, *PT* Portuguese, *n.a.* Not available

^a^Source: Census 2011 [25]. ^b^Source: 2005/2006 Portuguese National Health Survey [26]. ^c^Source: EQ-5D-3L Portuguese population norms [22]

The mean EQ-5D-5L index was lower than the EQ-5D-3L for all samples, as was expected. Given the youngness of the samples, it was also expected a higher EQ-5D-3L index and EQ-5D VAS when compared with the values of the Portuguese population.

The respondents that were interviewed individually were healthy individuals, whose minor health issues were related to allergies, asthma and one had had a knee injury in his preteens.

### Feasibility

The completion rate of the EQ-5D-5L was higher (99.5 %) than those of the EQ-5D-3L (93.0 %) in the respondents aged **≤** 30 years, and these results were consistent across the samples (sample aged > 30: 99.3 %-5L; 94.4 %-3L; overall sample: 99.5 %-5L; 96.1 %-3L). However the completion rates were different across dimensions (Table 2).

**Table 2:** Missing values by dimension

	Sample aged ≤30 (*n* = 624)		Sample aged >30 (*n* = 303)		Overall sample (*n* = 927)	
Dimension	EQ-5D-3L	EQ-5D-5L	EQ-5D-3L	EQ-5D-5L	EQ-5D-3L	EQ-5D-5L
Mobility	2 (0.3 %)	2 (0.3 %)	3 (1.0 %)	0 (0.0 %)	5 (0.5 %)	2 (0.2 %)
Self-care	4 (0.6 %)	2 (0.3 %)	8 (2.6 %)	0 (0.0 %)	12 (1.3 %)	2 (0.2 %)
Usual Activities	5 (0.8 %)	1 (0.2 %)	5 (1.7 %)	1 (0.3 %)	10 (1.1 %)	2 (0.2 %)
Pain/Discomfort	10 (1.6 %)	1 (0.2 %)	6 (2.0 %)	0 (0.0 %)	16 (1.7 %)	1 (0.1 %)
Anxiety/Depression	8 (1.3 %)	1 (0.2 %)	9 (3.0 %)	1 (0.3 %)	17 (1.8 %)	2 (0.2 %)

The results show that, although both instruments showed a good feasibility, the EQ-5D-5L seems to be “more feasible” than the EQ-5D-3L: while missing values ranged from 2 for mobility (0.3 %) to 10 for pain/discomfort (1.3 %) for the EQ-5D-3L, for the EQ-5D-5L missing values ranged from 1 for usual activities, pain/discomfort and anxiety/depression (0.2 %) to 2 for the other dimensions (0.3 %) for 5L. Missing values were on average 0.2 % (1.4) for the EQ-5D-5L and 0.9 % (5.8) for the EQ-5D-3L, indicating good feasibility for both instruments. The results were similar for the sample of respondents aged more than 30 years (0.1 % for the EQ-5D-5L and 2.0 % for the EQ-5D-3L) and for the overall sample (0.6 % for the EQ-5D-5L and 4.0 % for the EQ-5D-3L). In terms of the indexes, 602 (96.5 %) respondents aged up to 31 years old completed all five EQ-5D questions and therefore 5L and 3L indexes were computed only for these respondents. Similarly 5L and 3L indexes were computed for 285 (94.1 %) of the respondents aged more than 30 years old and for 887 (95.6 %) respondents of the overall sample.

### Inconsistency

Distributions of individuals’ responses across the EQ-5D-3L and EQ-5D-5L dimensions (cross tabulation of responses) are presented in Table 3. The results show that participants aged 30 years or under reported used all new five-scale levels of health within each of the EQ-5D dimensions. Inconsistent responses are marked in bold. The dimensions anxiety/depression (8) and mobility (4) presented the higher number of inconsistencies whereas the dimensions self-care and usual activities presented the lowest (0 and 1, respectively). The proportion of inconsistencies ranged from 1.3 % for anxiety/depression to 0.2 % for usual activities, whilst the average size of inconsistency was highest (2.3) for mobility and lowest (1.0) for usual activities, pain/discomfort and anxiety/depression (Table 3).

**Table 3:** Distributions of individuals’ responses across the EQ-5D-3L and EQ-5D-5L dimensions (sample aged ≤30), rank correlations and a summary of inconsistencies

Mobility							Self-care						
	5L							5L					
3L	1	2	3	4	5	Σ	3L	1	2	3	4	5	Σ
1	597	6	**0**	**1**	**2**	606	1	615	0	**0**	**0**	**0**	615
2	**1**	10	2	1	**0**	14	2	**0**	2	1	0	**0**	3
3	**0**	**0**	**0**	0	0	0	3	**0**	**0**	**0**	0	0	0
Σ	598	16	2	2	2	620	Σ	615	2	1	0	0	618
Spearman	0.732*						Spearman	1.000*					
Usual Activities							Pain/Discomfort						
	5L							5L					
3L	1	2	3	4	5	Σ	3L	1	2	3	4	5	Σ
1	574	12	**0**	**0**	**0**	586	1	424	68	**0**	**0**	**0**	492
2	**1**	26	2	3	**0**	32	2	**3**	100	16	1	**0**	120
3	**0**	**0**	**0**	0	0	0	3	**0**	**0**	**0**	0	1	1
Σ	575	38	2	3	0	618	Σ	427	168	16	1	1	613
Spearman	0.828*						Spearman	0.739*					
Anxiety/Depression							Summary of inconsistencies						
	5L						Dimension	n° (%)					Average
3L	1	2	3	4	5	Σ	Mobility	4 (0.6 %)					2.3
1	374	65	**3**	**0**	**0**	442	Self-care	0 (0.0 %)					-
2	**4**	118	44	5	**1**	172	Usual Activities	1 (0.2 %)					1.0
3	**0**	**0**	**0**	1	0	1	Pain/Discomfort	3 (0.5 %)					1.0
Σ	378	183	47	6	1	615	Anxiety/Depression	8 (1.3 %)					1.0
Spearman	0.780*												

Inconsistent responses are marked in bold. *p < 0.01

### Ceiling effect

Table 4 reports the proportion of “no problem” responses on EQ-5D-3L to the EQ-5D-5L and the absolute and relative ceiling effect reduction. The results show that 62.1 % of the respondents aged less than 31 years reported no problems (full health) on the EQ-5D-3L and 46.4 % on the EQ-5D-5L on all dimensions, indicating an absolute reduction of 15.7 % and a relative reduction of 25.3 % (Table 4). The value is lower in the comparison sample in the EQ-5D-3L (58.7 %) and slightly lower in the overall sample, whilst in the EQ-5D-5L the value is almost equal (46.8 %; 46.5 %). The self-care dimension showed the highest ceiling effect and the anxiety/depression dimension showed the lowest. Compared to the 3L, the proportion of respondents reporting no problems decreased in both samples. However the decrease is more evident in dimensions pain/discomfort and anxiety/depression, which showed the highest relative reduction on ceiling effect (14.4 % and 14.7 %) (Table 4). Differences in the ceiling effect were statistically significant in all dimensions except in self-care. After excluding inconsistent response the results for the ceiling effect reduction were very similar to the results presented in Table 4.

**Table 4:** Proportion of “no problem” responses on EQ-5D-3L to the EQ-5D-5L and ceiling effect reduction

	Sample aged ≤30 (*n* = 624)			Sample aged >30 (*n* = 303)			Overall sample (*n* = 927)			Sample aged ≤30 (*n* = 624)		Sample aged >30 (*n* = 303)		Overall sample (*n* = 927)	
	3L	5L	*p* ^a^	3L	5L	*p* ^a^	3L	5L	*p* ^a^	Absolute	Relative (%)	Absolute	Relative (%)	Absolute	Relative (%)
FH	376 (62.1 %)	288 (46.4 %)	0.001	168 (58.7 %)	141 (46.8 %)	0.001	544 (61.1 %)	429 (46.5 %)	0.001	15.7 %	25.3 %	11.9 %	20.3 %	14.6 %	23.9 %
MO	608 (97.7 %)	600 (96.5 %)	0.021	286 (95.3 %)	285 (94.1 %)	0.250	894 (97.0 %)	885 (95.7 %)	0.003	1.2 %	1.2 %	1.2 %	1.3 %	1.3 %	1.3 %
SC	617 (99.5 %)	619 (99.5 %)	1.000	293 (99.3 %)	300 (99.0 %)	1.000	910 (99.5 %)	919 (99.4 %)	1.000	0.0 %	0.0 %	0.3 %	0.3 %	0.1 %	0.1 %
UA	587 (94.8 %)	580 (93.1 %)	0.003	281 (94.3 %)	274 (90.7 %)	0.013	868 (94.7 %)	854 (92.3 %)	0.001	1.7 %	1.8 %	3.6 %	3.8 %	2.4 %	2.5 %
PD	493 (80.3 %)	428 (68.7 %)	0.001	220 (74.1 %)	196 (64.7 %)	0.001	713 (78.3 %)	624 (67.4 %)	0.001	11.6 %	14.4 %	9.4 %	12.7 %	10.9 %	13.9 %
AD	443 (71.9 %)	382 (61.3 %)	0.001	216 (73.5 %)	190 (62.9 %)	0.001	659 (72.4 %)	572 (61.8 %)	0.001	10.6 %	14.7 %	10.6 %	14.4 %	10.6 %	14.6 %

^a^McNemar test. *FH* Full health, *MO* Mobility, *SC* Self-care, *UA* Usual activities, *PD* Pain/discomfort, *AD* Anxiety/depression, 3 L-EQ-5D-3L, 5 L-EQ-5D-5L, *p*-*p*-value

It is worth noting that the mode was 1.0 for both indices in all the samples. However, the ceiling effect is more evident in the EQ-5D-3L, since there are more than 50 % of the respondents with an index of 1.0; while 50 % of the sample aged less than 31 years had an EQ-5D-5L index of 0.879.

### Level of agreement

The level of agreement between the dimensions of the EQ-5D-5L and 3L was accessed using Spearman’s correlation coefficient. The results show that the dimensions of the measures were strongly correlated, as was expected (Table 3), and these results were similar to what was observed in the comparison sample for dimensions self-care (1.000), pain/discomfort (0.788), anxiety/depression (0.802). For the dimension mobility (0.905) the correlation was higher in the comparison sample and lower in the dimension usual activities (0.690).

**Fig. 1:**
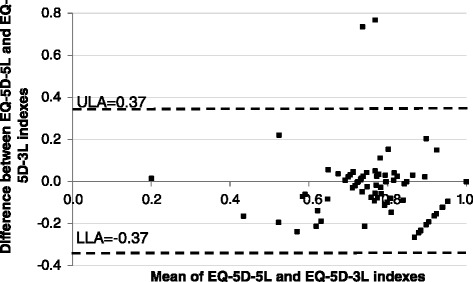
Bland-Altman plots (sample aged ≤30). ULA Upper limit agreement; LLA Lower limit agreement

The EQ-5D-5L and 3L scores for the 602 respondents aged ≤30 years old who completed both measures were strongly correlated, with a Pearson’s correlation coefficient of *r* = 0.760 (*p* < 0.001) and an ICC of 0.759 (*p* < 0.001). Similar results were observed in the comparison sample (0.758; 0.752) and in the overall sample (0.763; 0.762), where (*r*; ICC). Additionally the Bland-Altman plots (Fig. 1) also indicate a strong agreement between the EQ-5D-5L and 3L (only 4 % observations are beyond the limits of agreement). In what concerns the comparison sample, the number of observations beyond the limits of agreement is slightly higher (6.3 %). These results are consistent with those of Kim and colleagues [15].

### Known-groups validity

Non-parametric tests for respondents aged up to 31 years old showed significant results for both indexes by gender, health condition, labor situation and marital status (*p* < 0.001 for all situations), meaning that both measures were able to discriminate between the socio-demographic groups in analysis. We found similar results in the other samples.

### Discriminative properties

Table 5 displays the results of the area under the ROC curves, calculated to evaluate the performance of the EQ-5D-5L and the EQ-5D-3L indices in the identification of differences in individual health states.

**Table 5:** 1Area under receiver operating characteristic curves (AUC) with 95 % confidence intervals and comparability tests (sample aged ≤30)

	EQ-5D-5L		EQ-5D-3L			
Reported medical conditions	AUC	95 % CI	AUC	95 % CI	*χ* ^2^	*p*
None versus 1 or more conditions	0.382	0.323;0.441	0.383	0.328; 0.438	0.00	0.946
None or 1 condition versus 2 or more conditions	0.274	0.164; 0.383	0.270	0.168; 0.372	0.02	0.892

*95 % CI* 95 % confidence intervals

Looking at first row of Table 5, it is evident that the area under the ROC curve is almost the same for both measures. Similar results are shown when using the cut-off none or one medical condition versus two or more medical conditions. However the indices do not present statistically significant differences in their discriminatory capability.

### Content and linguistic analysis of explorative interviews

The explorative part of this study used cognitive interviews based on verbal probing to elicit respondents’ understanding and perceptions of the items and responses that make up the questionnaires designed to measure the EQ-5D-3L and the EQ-5D-5L, with a particular emphasis on the more recent 5L questionnaire. Analysis is based on CODA approach [24] and discourse analysis linguistic categories at the syntactic-semantic level [27]. Interview transcripts were carefully read and categorized according to each questionnaire item. Next, we focused on linguistic features which indicated different levels of certainty. Tables 6 and 7 list sample quotes from interviews. Table 6 illustrates each of the questionnaire’s dimensions and levels of severity mentioned in the interviews which were prone to low to moderate levels of certainty when conceptualizing differences between severity levels, and which indicated difficulty in verbalizing differences or in finding illustrative examples. The column “sample quotes” exhibits how the interviewees verbally represent and differentiate the dimensions; the column “linguistic extracts” portrays sample occurrences produced by the respondents which can be analyzed by looking at specific linguistic features produced in natural occurring discourse (lexical items, such as adjectives, adverbs, pronouns, voice, modality) for that particular questionnaire item, and which indicate various levels of uncertainty/certainty when interpreting levels of severity. The analysis of these discourse properties enable us to ascribe (covert) meaning to what is being stated (e.g. lexical items such as *maybe* or *I don’t know* or the use of modal verbs in parts of sentences such as ‘*I would say that’* indicate moderate levels of certainty). Particularly problematic areas were the distinction between the adjective pairs slight/moderate in the dimensions mobility, self-care and usual activities. However, distinctions between the dimension pairs pain/discomfort and anxiety/depression were less problematic, and presented linguistic features indicating high level of certainty and choice of specific lexical items (Table 7).

**Table 6:** Content analysis and linguistic analysis of interviews according to dimensions and levels slight, moderate and severe^a^

Dimensions & levels of severity		Sample quotes (content analysis)	Linguistic extracts	Linguistic features
Mobility	slight	*Minimal problems*	*Maybe, I don’t know*	Illustrations, moderate level of certainty
		*Pain is not strong*	*For example*	
		*Someone who limps*	*I would say that*	
		*I sprayed my ankle, but I can still walk*	*I don’t see any difference between moderate and severe*	
			*Something like that*	
	moderate	*Sprayed an ankle and need a clutch*	*A moderate problem*	Repetition
		*His problem is slightly more severe, for example instead of limping the leg is paralyzed*	*For example*	Illustration
			*Moderate is a bit more*…[does not complete sentence]	
Self-care	slight	*People who have problems in washing their teeth*	[long pause and difficulties in distinguishing slight and moderate]	Choice of imprecise qualifiers
		*Takes the toothbrush up to the mouth*	*To move is more complicated*	
	moderate	*They may need external help*	*May need*	Low level of certainty
Usual activities	slight	*Tiredness*		Imprecise lexical choice
		*Can practically do everything*	*Can practically do everything*	Generic pronouns
	moderate	*Some kind of deficiency*	*Maybe, possibly*	Modalization
		*Is able to do some things*		Some degree of certainty
Pain/Discomfort	Slight	*Something that we can easily change*		Illustration
		*It’s like a itch, we feel but we don’t give it too much thought*		High level of certainty
		*I feel some discomfort right now ‘cause of my allergies*		
	moderate	*Some kind of illness, the flu*	*[no occurrence of modals, and mitigators]*	High level of certainty
		*We feel it is hurting in a given place*		
		*Slight [discomfort] is more superficial it’s not so exaggerated*		
		*Moderate is like the name indicates the person can do some things and others can’t*		
Anxiety/Depression	slight	*I’m slightly [anxious] during [school] tests*	*I’m slightly [anxious] during [school] tests*	High level of certainty
		*A person has the notion s/he is anxious but it does not impacts on the way s/he acts and speaks*	*I’m slightly anxious to know my grade*	Choice of lexical nouns specific
		*When we’re going to take a harder [school] test*		
	moderate	*On the day of the test I’m moderately anxious, it’s not severe*	*On the day of the test I’m moderately anxious may start to stutter*	High degree of certainty
		*A moderately anxious person may start to stutter*		Illustration with own feelings and own context
				Choice of specific lexical items
	severe	*An extremely anxious person might even I don’t know have panic attacks*	*might even I don’t know*	Moderate to high level of certainty
				Choice of specific lexical items
		*The person shuts himself/herself at home doesn’t want to see anyone thinks only of committing suicide*		

^a^Interviews were conducted in Portuguese and extracts were translated by the authors and validated by an English native speaker fluent in Portuguese

**Table 7:** Distinctions between pairs of concepts

Items	Content analysis^a^	Linguistic extracts^a^	Linguistic features
Pain/Discomfort	*Association to physical pain and not other type of pain* [1–4]	*I think pain is something that pains us and discomfort is that we don’t feel 100 %* [3]	Choice of generic lexical items and indefinite pronouns
	*To measure the degree of pain is very difficult* [2]	*For me pain is to feel some pain, right? Discomfort is to feel bothered with something but it’s not really pain* [4]	High level of certainty
Anxiety/Depression	*These are totally different things* [1]	*When I’m depressed I’m sad, I don’t feel like doing anything*	First person discourse
	*These are two distinct things* [3]	*I think there are various types of anxiety but there’s only one type of depression* [2]	Choice of specific lexical items
	*These are two different issues* [4]	*I think these are two distinct things* [3, 4]	High level of certainty

^a^Numbers between square brackets indicate individual respondents

## Discussion

This paper compares the psychometric properties of the EQ-5D-5L with the EQ-5D-3L in a sample of young adults, aged 30 years or under, given that, to our knowledge, there has been no assessment of measurement properties or validation of the EQ-5D-5L in young adults.

The results show that, although both instruments showed a good feasibility, the EQ-5D-5L had a higher completion rate than the EQ-5D-3L, which is consistent with other studies [16]. The proportion of inconsistencies among the respondents aged less than 31 years averaged 0.7 % and this was significantly lower than what was reported in previous studies [15–17, 19]. Our findings show that participants used all new five-scale levels within each of the EQ-5D dimensions. These results are similar to those reported by Janssen and colleagues [17].

We expected a lower ceiling effect in the EQ-5D-5L and this hypothesis was verified. There was indeed a significant reduction in the ceiling effect. Compared to the 3L, the proportion of respondents reporting no problems decreased in both samples. However this decrease is more evident in the youngest sample. The reduction of the ceiling effect was higher in dimensions pain/discomfort and anxiety/depression and differences were statistically significant in almost every dimension. Although the decrease in the ceiling effect also occurred in other studies [15–17, 19], in this study the reduction was significantly higher and similar to what was found for a student Polish cohort [17], and these findings support the general idea that the EQ-5D-5L is an adequate measure of the HRQoL in young and relatively healthy adults.

The assessment of the level of agreement between the EQ-5D-3L and the EQ-5D-5L proved to be strong between the measures and these results are consistent with those of Kim and colleagues [15].

Known-groups validity was confirmed for both indexes by gender, health condition, labor situation and marital status for respondents aged up to 31 years old. Similar results were found for the other sample.

Regarding the discriminative properties of the measures, our findings show similar results for both of them. Indeed, it was found that the slightly EQ-5D-5L has a slightly better ability to discriminate between respondents with none or one medical condition from those with two or more medical conditions. However the indices do not present statistically significant differences in their discriminatory capability.

Explorative interviews indicated ambiguity and low degree of certainty in regards to conceptualizing differences between levels moderate-slight across three dimensions.

The findings of the present study provide evidence of the validity of the EQ-5D-5L in a sample of young adults (≤30 years). However a number of limitations should be considered when interpreting these findings. First, not all measurement properties were tested in the current study. We have followed part of the methodology used in previous studies, but we were not able to compute indexes used by other authors to assess the discriminatory power [15–17, 19], such as the Shannon index and the Shannon Eveness index, since the estimation of the first is applied for each dimension and needs the computation of a logarithm of the proportion of observations in the i*th* level. Given that our sample was relatively young and respondents did not state to have extreme problems in some dimensions, it was not possible to compute the logarithm for the dimensions in which there were no responses in at least one level. One further limitation was the non-randomness of the sample and its specific characteristics which mean it is not representative of the Portuguese population (e.g. women and single individuals were overrepresented). However, this does not constitute a real drawback for this study, since we strongly believe that although women and single individuals are overweighed in the sample this does not have a significant impact on the conclusions of the study given its aim. In fact, given that we aimed at comparing the performance of both measures in a sample of young adults (≤30 years), we were expecting some characteristics to be overrepresented. Nevertheless, the non-randomness of the sample implies that these results should be seen as sample results and conclusions cannot be drawn for the entire Portuguese population of young adults (≤30 years). Furthermore, when collecting the data, we followed a study design similar to other studies [15–17, 19], meaning that the EQ-5D-5L was always applied first, and there could possibly be an order effect. Moreover, the higher response rate for the EQ-5D-5L might partially be due to the fact that the 5L was administered first. In the explorative component of this study we have interviewed four students and applied a systematic analysis of both content and linguistic choices and patterns. These methods were used as a complementary analysis and therefore the number of interviews was acceptable in cognitive terms, however we recommend more interviews, as these would have enriched the content of the paper and we will proceed accordingly in the future.

In future studies, the properties of the EQ-5D-5L should be further examined in random samples of healthy and/or young individuals. Further research on the validity, reliability and responsiveness of the EQ-5D-5L on the general population and in different patients’ settings is also needed. Furthermore, drawing from the explorative interviews, it is highly recommended that both a content-based analysis of language data (suitable for highlighting the conscious process that participants verbalize) linked to the analysis of the structure and linguistic choices involved in these verbalizations contain rich information that is worth exploring in future research.

## Conclusions

In light of the properties analyzed, the EQ-5D-5L performed better than the EQ-5D-3L. These results show that this new version contributed to a significant reduction in the ceiling effect which was one of the most relevant limitations of the 3-level EQ-5D. However, even though the ceiling effect is reduced, the explorative study reflects on the methodological issues that arise when questionnaires are used, namely about how questions are linguistically framed (namely subjectivity in interpreting slight vs moderate and the noun pairs pain/discomfort and anxiety/depression) and if this instrument is adequate for young healthy adults. Therefore, these findings need to be replicated in other samples of healthy and sick individuals. Further research is also needed to fully understand the role of the different layouts in the respondents’ answers.
